# Antioxidant, anti-inflammatory and antimicrobial activity of natural products in periodontal disease: a comprehensive review

**DOI:** 10.3389/fbioe.2023.1226907

**Published:** 2023-08-03

**Authors:** Nansi López-Valverde, Antonio López-Valverde, Javier Montero, Cinthia Rodríguez, Bruno Macedo de Sousa, Juan Manuel Aragoneses

**Affiliations:** ^1^ Department of Medicine and Medical Specialties, Faculty of Health Sciences, Universidad Alcalá de Henares, Madrid, Spain; ^2^ Instituto de Investigación Biomédica de Salamanca (IBSAL), Madrid, Spain; ^3^ Department of Surgery, Instituto de Investigación Biomédica de Salamanca (IBSAL), University of Salamanca, Salamanca, Spain; ^4^ Department of Dentistry, Universidad Federico Henríquez y Carvajal, Santo Domingo, Dominican Republic; ^5^ Institute for Occlusion and Orofacial Pain Faculty of Medicine, University of Coimbra, Coimbra, Portugal; ^6^ Faculty of Dentistry, Universidad Alfonso X El Sabio, Madrid, Spain

**Keywords:** periodontal disease, natural products, oxidative stress, anti-inflammatory activity, antimicrobial activity, natural antioxidant therapy

## Abstract

Periodontal diseases (PD) are common chronic inflammatory oral pathologies that are strongly linked to others not found in the mouth cavity. The immune system mediates the host response, which includes the upregulation of proinflammatory cytokines, metalloproteinases, and reactive oxygen species (ROS); the latter may play an important role in the establishment and progression of inflammatory diseases, particularly periodontal disease, via the development of oxidative stress (OS). Natural antioxidants have powerful anti-inflammatory properties, and some can reduce serum levels of key PD indicators such tumor necrosis factor (TNF) and interleukin IL-1. This review compiles, through a thorough literature analysis, the antioxidant, anti-inflammatory, and antibacterial effects of a variety of natural products, as well as their therapeutic potential in the treatment of PD.

## 1 Introduction

Periodontal diseases (gingivitis and periodontitis) (PD) are chronic inflammatory polymicrobial pathologies linked with biofilm that harm the tooth’s supporting tissues and cause tooth loss, as well as related bone lesions (Highfield, 2009).

Its clinical diagnosis is based on visual and radiographic inspection, which demonstrates the presence of symptomatology in periodontal tissues, indicating the presence of the illness (Pihlstrom et al., 2005). Apart from inflammation or gingival edema, there is frequently some spontaneous or prompted bleeding in chronic gingivitis; however, chronic periodontitis has a more extensive symptomatology, including mobility, tooth displacement, halitosis, and recurrent periodontal abscesses (Armitage, 2004). In the early stages, there is frequently a vertical pattern of bone degradation in the first molars and a horizontal pattern in the incisors (owing to the thickness of the alveolar bone); in the advanced stages, bone loss may be universal and display a single horizontal pattern (Albandar, 2014).

They are the sixth most common pathology in the world, affecting approximately 750 million people, and are the leading cause of tooth loss, resulting in masticatory dysfunction that affects patients’ quality of life, nutrition, and self-esteem, resulting in a high health cost and economic impact on the various health systems (Jin, 2013; Chapple et al., 2015).

Oxidative stress (OS) is defined as an imbalance between free radical generation and antioxidant systems that arises when the recipient organism is unable to correct for an excess of reactive oxygen species (ROS) (Kopáni et al., 2006). There is growing evidence that ROS play a role in chronic inflammatory pathologies such as PD; thus, the inflammatory effects of these pathologies could be controlled by using antioxidant compounds, and oxidative stress could be a therapeutic target for their treatment (Bhattarai et al., 2017; Sczepanik et al., 2020).

Certain natural antioxidants have been found to be effective in the treatment of periodontitis, as they reduce inflammation and enhance the body’s antioxidant defense system; antioxidant-rich diets have been shown to have a strong anti-inflammatory capacity (Arora et al., 2013; Belludi et al., 2013). Some studies have found a significant reduction in serum levels of tumor necrosis factor (TNF) and interleukin IL-1 (two critical biomarkers of periodontitis) in people with chronic periodontitis after consuming tomato drinks for a certain time (Riso et al., 2006). Some flavonoids, such as resveratrol, have been shown to be able to slow the progression of periodontal disease (Bhattarai et al., 2016). Others, such as turmeric and quercetin, have also been shown in preclinical research to be effective against crestal bone loss (Guimaraes et al., 2011; Zhou et al., 2013; Tamaki et al., 2014; Correa et al., 2017).

The present study sought to evaluate the antioxidant, anti-inflammatory and antibacterial benefits of a variety of natural products, as well as their therapeutic potential in PD, in addition to standard therapy.

## 2 Periodontal diseases and oxidative stress

There is mounting evidence that certain inflammatory diseases are the outcome of OS caused by ROS. OS generates an imbalance between ROS and the organic antioxidant system, which can result in DNA, protein, and lipid damage (Hussain et al., 2016).

Inflammation is a protective mechanism associated with infection, and periodontal disorders are considered inflammatory illnesses capable of generating diverse protein oxidations and thus elevated ROS (Su et al., 2009). Some researchers have investigated the link between oxidative stress and inflammation, highlighting the harmful significance of OS in chronic inflammatory disorders. Sculley and Langley-Evans evaluated total salivary antioxidant capacity in 129 patients and concluded that periodontal disease is related with decreased salivary antioxidant status and increased oxidative damage in the oral cavity (Sculley and Langley-Evans, 2003; Saygun et al., 2011). Uric acid is the most common antioxidant found in saliva; a study of 129 subjects in which periodontal health was assessed using the Periodontal Community Index of Treatment Needs revealed a direct relationship between saliva uric acid levels and periodontal treatment needs of patients with periodontal disease; thus, individuals with elevated saliva uric acid concentrations decrease Periodontal Community Indexes of Treatment Needs, relative to individuals with normal saliva uric acid concentrations. High levels of albumin in saliva have also been linked to periodontal health, while low levels have been linked to periodontal deterioration (Sculley and Langley-Evans, 2003; Ogawa et al., 2006; Iwasaki et al., 2008). Because saliva is in intimate contact with oral tissues, it can reflect physiological, pathological, and molecular changes that occur in them.

Excessive generation of ROS can induce irreversible cell damage and ultimately cell death by necrosis and apoptosis (Kohan et al., 2020). One of the main pathogens causing PD is the gram-negative anaerobic bacterium *Porphyromona gingivalis*, which has been linked to cerebrovascular lesions leading to pathologies such as stroke and Alzheimer’s disease through disruption of the blood-brain barrier (Ide et al., 2016).

**FIGURE 1:**
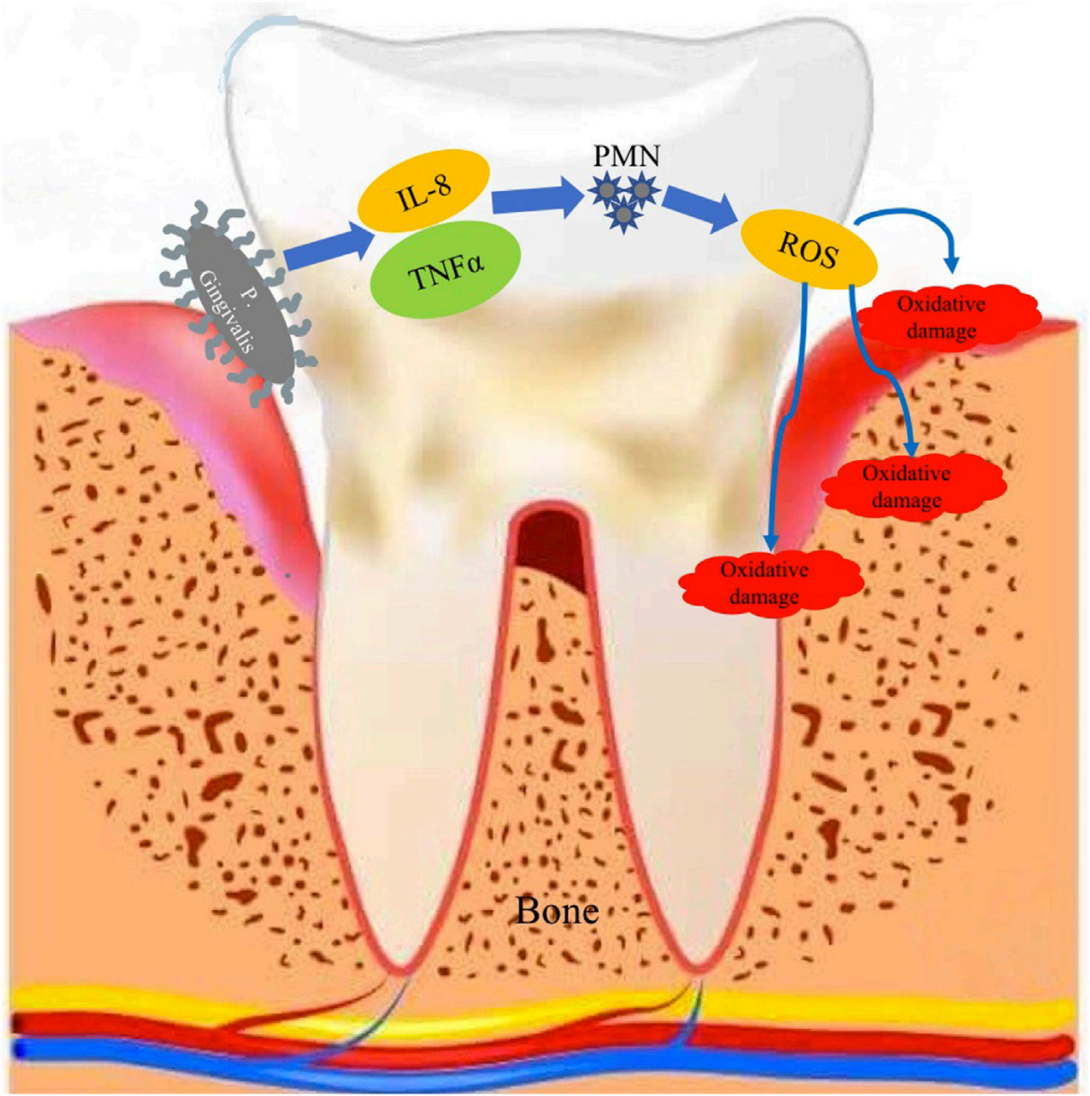
Schematic representation of the release of IL-8 and TNFα, following the destruction of connective tissue and bone around the tooth by *Porphyromona gingivalis* and the increased activity of PMNs in the production of ROS and their impact on the supporting apparatus of the tooth.

Sculley and Langley-Evans demonstrated that the bacterium *Porphyromona gingivalis*, which destroys the connective tissue and bone around the tooth root, causes the release of interleukin 8 (IL-8) and TNFα, leading to an increase in the number and activity of polymorphonuclears (PMNs), which cause the production of ROS as a host response to infection. As a result, PD patients have an increased number and activity of PMNs, as well as a high level of ROS release, leading to increased oxidative damage to gingival tissue, periodontal ligament and alveolar bone (Sculley and Langley-Evans, 2002). A specific PMN phenotype has also been shown to play a certain role in the development of periodontitis (Dias et al., 2011) (Figure 1).

Pathogenic bacteria associated in the pathogenesis of PD [*Porphyromona gingivalis, Treponema denticola*, and *Tannerella forsythia* (Hajishengallis, 2014)], particularly *Porphyromona gingivalis*, affect host defenses, changing the formation and evolution of the bacterial community under homeostatic settings. When pathogenic bacteria cause an inflammatory response, the organism secretes anti-infection chemicals such as cytokines, metalloproteinases, prostaglandins, and proteolytic enzymes through inflammatory cells. Tissue damage is caused by the stimulation of proinflammatory cytokines ((IL)-1β, IL-6, IL-8, IL-12, IL-17), TNF-α, and nuclear factor kappa B receptor activating ligand, as nuclear factor kappa-B receptor activating ligand, interleukins (IL)-1β, IL-6, tumor necrosis factor- (TNF-α), and prostaglandin E2 regulate osteoclastogenesis (Martínez-García and Hernández-Lemus, 2021).

Glutathione is an intracellular antioxidant tripeptide (γ-glutamyl-cysteinyl-glycine), and its deficiency causes increased ROS generation, inflammation, an imbalanced immunological response, and increased susceptibility to infection (Biswas and Rahman, 2009). Glutathione peroxidases (GPx) are crucial antioxidants in the fight against oxidative stress; Patel et al. (2009), Patel et al. (2012) established in clinical trials the role of eGPx as a marker of oxidative stress in PD and that GPx might be considered a marker of OS in PD. Enzymatic antioxidants, on the other hand, include glutathione reductase and catalase, though additional research is needed to explore the changes in these antioxidants during the periodontitis process. Because of their short lifetime, ROS are difficult to detect, and the related waste products, as well as the activity of non-enzymatic and enzymatic antioxidants, are considered useful markers in the evaluation of OS in PD (Chen et al., 2019).

## 3 Use of natural antioxidants in the therapy of periodontal diseases

Traditional medicine is worth a trillion dollars and is part of the expanding global pharmaceutical industry of health, wellness, and beauty, but more than 40% of the formulations are based on natural products; it has been estimated that the global economic impact of using phytotherapeutic products in the treatment of various pathologies, exceeds 100 million dollars annually (World Health Organization, 2019).

**FIGURE 2:**
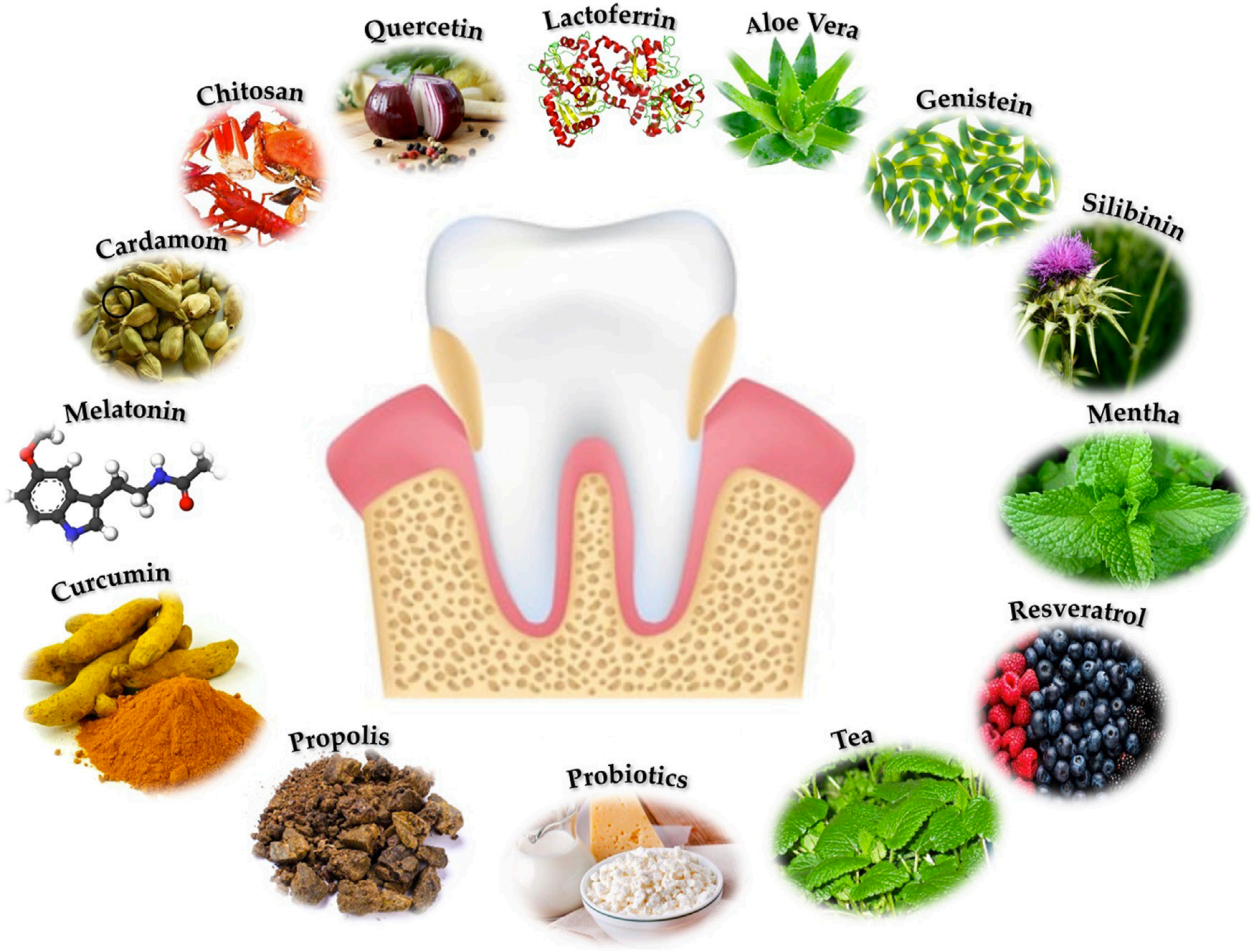
Most common natural products used in the treatment of PD.

In comparison to commonly used medicines, natural products have a broad spectrum of action against ROS mediators and a good margin of safety and tolerance (Isola et al., 2019), and the essential role that natural antioxidant products, can play in both the treatment and prevention of PD has been proven (Isola, 2020; Janakiram et al., 2020; Laleman and Teughels, 2020). For all of these reasons, we examined the role of natural products in the treatment of periodontal disease in this review, which we divided into eight categories: molecules, plants, flavonoids, phytonutrients, microorganisms, proteins, compound products, and phytohormones (Table 1) (Figure 2).

**TABLE 1:** Categorization of the natural products analyzed.

Product category	Product
Molecules	Chitosan
Plants (phytotherapy)	Cardamon
	Curcumin
	Tea
	Mentha
	Aloe Vera
Flavonoids	Quercetin
	Genistein
	Silibinin
Phytonutrients	Resveratrol
Microorganisms	Probiotics
Proteins	Lactoferrin
Compound products	Propolis
Phytohormones	Phytomelatonin

### 3.1 Molecules

#### 3.1.1 Chitosan

Chitosan is a natural polysaccharide formed from the partial deacetylation of chitin, which is found in the exoskeletons of crustaceans and some fungi. It is soluble in acidic conditions, and its high viscosity and biocompatibility, as well as its capacity to retain water, make it excellent for usage in a variety of forms, including membranes, gels, sponges, and so on (Abd El-Hack et al., 2020). It has a high antibacterial and antifungal activity, depending on the pathogen, concentration, and pH of the medium; this element is critical for its antimicrobial activity, as it acts only in pH 6.5 media (Sahariah and Másson, 2017; Wei et al., 2019; Qin and Li, 2020). It has antibacterial activity against both Gram-positive and Gram-negative bacteria, albeit its efficiency against one or the other is debatable (Hosseinnejad and Jafari, 2016; Ardean et al., 2021). Costa et al. (2014) studied *in vitro* the antimicrobial effect of chitosan on five biofilm-forming periodontal pathogens (*Porphyromonas gingivalis, Prevotella intermedia, Prevotella buccae, Tanerella forsythensis* and *Aggregatibacter actinomycetemcomitans*) and found that chitosan showed a strong effect against periodontal path-ogens through interference in bacterial co-aggregation, by inhibiting *Clostridium vilaceum* violacein, thus inhibiting biofilm formation. *In vitro*, Arancibia et al. (2013) found that chitosan particles can limit the growth of *Porphyromonas gingivalis* and *Aggregatibacter actinomycetemcomitans* as well as regulate the inflammatory response of human gingival fibroblasts.

Its antioxidant action is not its main characteristic, although it can be increased with synthetic products. Sun et al. (2007) found that antioxidant activity increases in inverse proportion to molecular weight (MW), and that high MW chitosan’s lack antioxidant action. Curcio et al. investigated the antioxidant capacity of chitosan functionalized with gallic acid and catechin and found a considerable improvement in ROS inhibition (Curcio et al., 2009). In general, studies show no or little antioxidant activity of chitosan, but all agree on the enhancing effect of this activity, through the insertion of phenolic chemicals into the chitosan backbone (Abd El-Hack et al., 2020). Despite this, many antioxidants encounter numerous limitations, such as instability and rapid destruction (Yang et al., 2016). To address these problems, researchers are exploring the possibility of grafting antioxidants onto chitosan, in particular polyphenols and other acids such as ferulic and chlorogenic acids, which have become of great medicinal importance (Lunkov et al., 2020; Zhao et al., 2022).

### 3.2 Plants (phytotherapy)

#### 3.2.1 Cardamom

The fragrant plant known as cardamom (Elettaria cardamomum) is grown in a number of nations throughout Asia, Africa, and South America. It is utilized as a culinary spice and is regarded as a significant source of terpenoids, alkaloids, and phenolic compounds (Noumi et al., 2018). According to published data, the essential oils it contains, have potential applications as an antibacterial, against Gram-positive and Gram-negative pathogens, as an antioxidant, and as a bacterial inhibitor; it also has anti-inflammatory properties (Bhattacharjee and Chatterjee, 2013; Cui et al., 2020).

Souissi et al. (2020) investigated the antibacterial activity of cardamom against the main periodontal pathogens (*Aggregatibacter actinomycetemcomitans, Fusobacterium nucleatum, Porphyromonas gingivalis* and *Prevotella intermedia*) and found that cardamom extracts inhibited biofilm formation and significantly decreased the secretion of IL-1β, TNF-α and IL-8 by macrophages, concluding that cardamom extracts would be of therapeutic interest against periodontal infections. Singh et al. (2008) discovered that cardamom essential oil and cardamom with a high terpene concentration, may contribute to its antioxidant properties. Mamgain et al. (2017) examined the efficacy of cardamom and chlorhexidine in the control of plaque and gingivitis in a randomized study of 60 patients, discovering a similar efficiency between both products.

#### 3.2.2 Curcumin

It is a polyphenol derived from the rhizome of Turmeric longa with numerous medicinal properties, capable of influencing the immune system (Gautam et al., 2007; Jagetia and Aggarwal, 2007). In a recent meta-analysis, Zhang et al. (2022a) evaluated the anti-inflammatory efficacy of curcumin as an adjunct to non-surgical periodontal treatment, concluding that it demonstrates anti-inflammatory efficacy in adjunctive periodontal disease treatment, reducing gingival indices and sulcus bleeding rates.

Curcumin’s usage is supported by preclinical research. Curylofo-Zotti et al. (2018) administered natural curcumin and chemically modified curcumin to rats; however, only the modified curcumin reduced osteoclasts; on the contrary, the natural curcumin group reduced the number of apoptotic cells in gingival tissues and osteocytes in the alveolar bone crest. In a similar experimental model, de Almeida Brandão et al. (2019) found that low doses of chemically modified curcumin taken orally, were sufficient to significantly inhibit alveolar bone resorption associated with experimental periodontal disease; additionally, inflammatory gene expression and macrophage phagocytosis were reduced *in vitro*, while ROS production was stimulated.

However, there are controversies in clinical trials regarding the adjuvant treatment of periodontitis with curcumin gel; Kaur et al. (2019) found a temporary reduction in inflammation but no statistically significant results, when compared to the group of patients treated exclusively with scaling and root planing. Similarly, Mohammad found a significant reduction of IL-11β and TNF-α in the group of patients with chronic periodontitis, treated by scaling and root planing and curcumin gel injection, in a comparative clinical study (Mohammad, 2020). In contrast, Pérez-Pacheco et al. (2021) found no improvement in clinical outcomes, nor differences in cytokine levels between the experimental groups, and *Aggregatibacter actinomycetemcomitans* counts in the groups increased only in the control.

#### 3.2.3 Tea

The *Camellia sinensis* plant produces three variations based on processing: green tea is made from unfermented leaves, oolong tea leaves are partially fermented, and black tea is fully fermented. Green tea, created from unfermented leaves, is regarded a strong antioxidant that strengthens and controls the immune system, whereas other types are prepared from partially or totally fermented leaves, losing most of its characteristics (Chacko et al., 2010). Green tea catechin may be beneficial in the prevention of periodontal disease. *In vitro* studies have revealed that green tea catechin suppresses the development of *Porphyromonas gingivalis* and *Prevotella intermedia*, two frequent infections in Parkinson’s disease, and that tea polyphenols limit the formation of harmful metabolites by *Porphyromonas gingivalis* (Kushiyama et al., 2009). In a study of the clinicomicrobiological effects of green tea on a sample of twenty patients, researchers discovered a substantial reduction in *Tannerella forsythus* and *Porphyromonas gingivalis* in the study group compared to the control group (Hattarki et al., 2013). Kudva et al. (2011) compared the use of locally administered green tea catechin, with scaling and root planing to scaling and root planing alone in the treatment of chronic periodontitis and found that local administration of green tea catechin, combined with scaling and root planing is more effective than scaling and root planing alone. Rezvani et al. (2021) evaluated changes in salivary IL-1 concentration in patients with chronic periodontitis after daily consumption of green tea, in a randomized study on a sample of thirty subjects and found a significant reduction in salivary IL-1 concentration in the experimental group, reporting that green tea supplementation, in addition to scaling and root planing, can temporarily reduce salivary IL-1 levels in patients with chronic periodontitis.

A recent meta-analysis of randomized controlled trials on the effects of green tea on gingivitis and periodontitis, concluded that it has benefits in these pathologies; however, they did not find enough evidence that green tea can completely replace chlorhexidine, so the latter remains the recommended solution for the local treatment of these processes (Mazur et al., 2021).

#### 3.2.4 Mentha

Mentha is a herbaceous plant genus containing 13 to 18 species in the *Lamiaceae* family. It is found in Europe, Asia, Africa, Oceania, and America. Some Mentha species, including *Mentha spicata*, *Mentha x piperita, Mentha arvensis, Mentha rotundifolia, Mentha suaveolens*, and *Mentha pulegium*, have been shown to have antimycobacterial and antioxidant activities (Diniz do Nascimento et al., 2020; Aldoghachi et al., 2021).

Ashrafi et al. (2019) studied the antibiofilm capacity of *Mentha piperita* essential oils in chitosan nanogel *in vitro*, and their findings suggest that they might be used as an antibiofilm agent in toothpaste or mouthwash formulations. Kumar et al. (2011) investigated the antibacterial activity of Mentha oil isolates against twelve bacterial strains and nine fungus strains, finding that the microorganisms were significantly inhibited. Silva et al. (2022) revealed *in vitro* that *Mentha piperita* essential oil has antibacterial effects against *Staphylococcus aureus* and *Listeria* monocytogenes, as well as significant antioxidant activity, blocking up to 73% of radicals.

We discovered no clinical trials that linked Mentha and PD; instead, Schönknecht et al. (2021) conducted a study of the efficacy of a herbal compound extract, of which it was a component, in the treatment of gingivitis and periodontitis.

#### 3.2.5 Aloe Vera

It is an Asphodelaceae cactus-like plant. With over 500 species, it is found in various parts of the world and is utilized in a variety of consumer items. It includes 75 active components, including vitamins, enzymes, minerals, sugars, ligins, saponins, salicylic acids, and amino acids, which have been demonstrated *in vitro* and *in vivo* to have anti-inflammatory, anti-arthritic, and antibacterial actions (Milutinovici et al., 2021). The majority of research look at its effect in toothpastes or mouthwashes, however others look at its mode of action in individuals with periodontitis. In a randomized study, Ashouri Moghaddam et al. (2017) assessed the effect of local application of aloe vera gel as an adjunctive treatment to scaling and root planing on a sample of 20 patients with chronic periodontitis, finding no differences between the experimental and control groups, despite the experimental group having significantly lower gingival indices and probing depth at the end of the study. Similarly, Pradeep et al. (2016) investigated the clinical efficacy of aloe vera gel as an adjunct to scaling and root planing in the treatment of type 2 diabetes mellitus and chronic periodontitis, finding a significantly higher gain in clinical attachment levels in the experimental group.

### 3.3 Flavonoids

#### 3.3.1 Quercetin

Quercetin is a flavonoid found in large amounts in both vegetables and fruits, such as tomatoes, onions, potatoes, apples, grapes, and broccoli, and is believed to be the most prevalent and abundant flavonoid in the human diet (Xu et al., 2019). It has been the subject of much research due to its anti-inflammatory, antioxidant, and anticarcinogenic qualities, and has been found to be a protective agent against oxidative damage to nerve cells, suggesting that it may be useful in the treatment of Alzheimer’s disease and PD (D’Andrea, 2015; Sharma et al., 2020; Singh and Kumar, 2018). It has also been shown to reduce alveolar bone resorption and limit the production of proinflammatory cytokines (Napimoga et al., 2013; Xiong et al., 2019). Wei et al. (2021) revealed *in vitro* and *in vivo* that quercetin would be an effective therapeutic agent for periodontitis due to its antioxidant capacity, its ability to alleviate oxidative damage to periodontal ligament cells, and its ability to prevent alveolar bone absorption. Mooney et al. (2021) conducted an *in vitro* and *in vivo* study to investigate the effect of orally administered quercetin on host inflammatory response, oral microbial composition and periodontal disease phenotype; they found a reduction in gingival cytokine expression, inflammatory cell infiltrate and alveolar bone loss; in addition, analysis of the microbiome revealed a healthier oral microbial composition in the quercetin-treated group compared to the control group, with a decrease in the number of pathogenic species such as *Enterococcus, Neisseria*, and *Pseudomonas* and an increase in the number of non-pathogenic *Streptococcus* and bacterial diversity. *In vitro*, quercetin reduced the generation of proinflammatory cytokines. Recent studies have explored the ability of quercetin on the osteogenic development of mesenchymal stem cells, obtained from human bone marrow (Bian et al., 2021), revealing that the effective non-cytotoxic dose would be 1–2 μm. He et al. (2020) revealed the potential of quercetin to inhibit the production of inflammatory mediators such as IL-1 β, IL-6, IL-8, and TNF-α in human gingival fibroblasts stimulated by lipopolysaccharide from *Porphyromonas gingivalis*. Other investigations have found extraordinary antibacterial action against *Porphyromonas gingivalis* biofilms, as well as significant discontinuous damage to their cell membrane, with varying concentrations of quercetin, with increasing severity with increasing concentration (Sehmisch et al., 2010a).

#### 3.3.2 Genistein

Genistein is an isoflavone that was initially discovered in 1899 from *Genista tinctoria* and chemically synthesized in 1928; it is abundant in soybean products and has been linked to improved bone quality and bone density (Sehmisch et al., 2010b). Bhattarai et al. (2017) investigated genistein’s anti-inflammatory, anti-osteoclastic, and antioxidant effects *in vivo* and discovered that it inhibits mitochondrial degradation and ROS formation via autophagy, which is important for mitochondrial health and cellular function. Other research has investigated the effect of this isoflavone in the loss of alveolar bone height and bone volume, in generated periodontitis *in vivo*, indicating that it might be utilized to treat human periodontitis in the future (Choi et al., 2016).

#### 3.3.3 Silibinin

It is a natural flavonoid, derived from the seeds of *Sylibum marianum* of the *Asteraceae* family, with anti-inflammatory and antioxidant effects (Bijak, 2017). Certain research has demonstrated its antioxidant capacity, evidenced by free radical scavenging and an increase in intracellular antioxidant enzyme levels, suggesting that its antioxidant and anti-inflammatory activities are related to its ability to influence the immune system (Federico and Loguercio, 2017). Found a reduction in alveolar bone loss and apoptosis of periodontal ligament cells, as well as a reduction in oxidative damage to lipids, proteins and DNA in the periodontal lesion zone, in an *in vivo* model of induced periodontitis in rats, and *in vitro* in a human periodontal ligament cell model; silibinin administration decreased ROS generation in the *in vitro* model. Furthermore, it acted as a potent anti-inflammatory in both *in vivo* and *in vitro* models by suppressing the production of inflammatory mediators (Li et al., 2023).

### 3.4 Phytonutrients

#### 3.4.1 Resveratrol

It is a natural phenol generated in numerous plants in response to pathogen assault; it is found in grapes and red fruits such as raspberries, blueberries, and blackberries (Zhang et al., 2021). It is thought to lower inflammation and OS, and some preclinical studies suggest that it may be effective in the prevention of PD development (Andrade et al., 2019). Several studies (Corrêa et al., 2019; Molez et al., 2020; Cirano et al., 2021) in experimental animal models, evaluated the efficacy of resveratrol on induced periodontitis and discovered reduced inflammation, lower levels of proinflammatory factors, and beneficial effects on alveolar bone loss, in the progression of experimental periodontitis.

Clinical trials support the use of resveratrol as an adjuvant therapy, in combination with non-surgical periodontal treatment. Zare Javid et al. (2017) discovered that using resveratrol as an adjunct to non-surgical periodontal therapy, resulted in considerably higher improvement, in a double-blind clinical study of 43 diabetic individuals with chronic periodontitis. In a major investigation on a large sample of 160 patients, Zhang et al. (2022b) revealed that resveratrol, reduces systemic local inflammatory indicators and systemic endotoxin, implying that 500 mg/day of resveratrol, would be the appropriate dose for individuals with periodontitis. Javid et al. (2019) conducted a study of inflammatory, antioxidant, and periodontal markers in patients with type 2 diabetes and PD, treated with resveratrol supplementation, indicating that daily resveratrol supplementation, would not change TNF-α and clinical attachment loss, but would be beneficial in lowering serum IL6 levels.

### 3.5 Microorganisms

#### 3.5.1 Probiotics

The World Health Organization (WHO) defines probiotics as bacteria that are beneficial to the host when used in appropriate proportions. They are live, non-pathogenic microorganisms that are capable of improving microbial balance (Williams, 2010). Probiotics have been extensively investigated as a host-modulating therapy in periodontitis (Nguyen et al., 2021; Amato et al., 2022; Gao et al., 2022). They act as anti-inflammatory in infected areas, with a large amount of *Porphyiromonas gingivalis* and a large component of proinflammatory cytokines, and lactobacilli have been shown to reduce the level of inflammatory cytokines in these areas; on the other hand, probiotic strains produce lactic acid, reuterin, reutericicline and bacteriocin and slow the growth of periodontal pathogens. (Moraes et al., 2020).

PDs produce a dysbiosis of the oral microbiome leading to an exaggerated immune response by the host and, consequently, destruction of dental supporting tissues. In order to counteract this dysbiosis, some studies have proposed the use of probiotics to restore an adequate microbiome to slow the progression of PD (Bizzini et al., 2012; Alkaya et al., 2017; Kuru et al., 2017). It is also known that species such as Bifidobacterium are able to reduce the adhesion of *Porphyiromonas gingivalis* and that *Bifidobacterium lactis* would be an antagonist of periodontal pathogens and could be used as an adjuvant in periodontal treatment (Argandoña Valdez et al., 2021); Oliveira et al. (2017) demonstrated *in vivo*, that topical subgingival use of Bifidobacterium lactis, promotes a protective effect against alveolar bone loss, by modifying immunoinflammatory and microbiological parameters.

Nevertheless, although there is a great profusion of studies in the literature, there is no unanimity among researchers to demonstrate its efficacy in the treatment of PD, and the certainty of probiotic therapy on periodontal health seems weak; on the other hand, there are no well-designed clinical trials with adequate follow-up. Therefore, the routine use of probiotics for these purposes, is currently unfounded (Farias da Cruz et al., 2022).

### 3.6 Proteins

#### 3.6.1 Lactoferrin

Lactoferrin is an iron-binding glycoprotein, a component of human secretions, including saliva and gingival crevicular fluids, synthesized by exocrine glands and neutrophils in areas of inflammation and infection. In addition to an important role in immune regulation, antimicrobial, antiviral, antioxidant, and cell growth modulating activities have been attributed to it. This depends on its ability to sequester iron necessary for bacterial growth and survival (Berlutti et al., 2011).

Kalmar and Arnold (1998) were the first to demonstrate that human lactoferrin was bactericidal against *Actinobacillus actinomycetemcomitans*, an important periodontal pathogen, suggesting that lactoferrin contributes to the elimination of this pathogen by human neutrophils and may play an important role in the innate secretory defense against this periodontopathogen. Wakabayashi et al. (2009) evaluated *in vitro* the effects of lactoferrin on the growth and biofilm formation of *Porphyromonas gingivalis* and *Prevotella intermedia*, demonstrating the potential usefulness of this glycoprotein for the prevention and treatment of PD and as an adjuvant therapy for PD. A single-center parallel-group comparative study conducted by Nakano et al. (2017) on a sample of 47 subjects over 65 years of age, reported that long-term ingestion of lactoferrin-containing tablets, promotes a shift in the subgingival plaque microbiota from a gram-negative to a gram-positive dominated community, which could contribute to significant improvements in gingival conditions. Ishikado et al. (2010) administered liposomal bovine lactoferrin orally for 4 weeks to a small sample of subjects with PD and evaluated probing depth, bleeding on probing, gingival crevicular fluid volume, and TNF-α, (IL)-1β and IL-6 levels.

They found that probing depth was significantly reduced with lactoferrin implementation, but bleeding on probing and gingival crevicular fluid volume did not change significantly; they also found a significant decrease in cytokine production. However, although Wakabayashi et al. (2010); Gruden and Poklar Ulrih (2021) in their reviews concluded that lactoferrin and its derived peptides, have a broad spectrum of antimicrobial activities, closely related to the amino acid compositions of the protein or peptide and appear to be inhibitors of periodontopathic bacterial biofilm *in vitro* and *in vivo*, certain studies have questioned their effect on the growth of *Actinobacillus actinomycetemcomitans* (Fine and Furgang, 2002).

The study of oxidative stress and the degree of periodontal inflammation by quantifying lactoferrin, in the gingivocrevicular fluid, was carried out by Yadav et al. (2014) by enzyme-linked immunosorbent assay on a sample of 50 subjects, divided into two groups of 25, according to gingival index, probing pocket depth, clinical attachment loss and alveolar bone loss, who underwent non-surgical periodontal treatment. They observed that lactoferrin levels were higher in the periodontitis group, compared to the healthy group and decreased with periodontal treatment; concluded that lactoferrin plays an important role in periodontal disease and that the quantification of crevicular lactoferrin, may be a marker for detecting periodontal inflammation and OS.

### 3.7 Compound products

#### 3.7.1 Propolis

Bee-derived products are considered a source of natural antioxidants capable of counteracting the effects of OS that accompanies the pathogenesis of various diseases. Propolis is a resinous mixture produced by honeybees by mixing their saliva with resinous substances collected mainly from flowers, leaves, stems and bark crevices of poplars, willows, birches, elms, alders, beeches, conifers and horse chestnuts (Šuran et al., 2021). In addition to its potent antioxidant effect, anti-inflammatory, antimicrobial, anticancer, analgesic, and immunomodulatory effects are attributed to it.

Different studies have investigated the role of propolis in the treatment of PD. Nakao et al. (2020) in a double-blind clinical trial, on a sample of 24 patients, studied the effect of propolis in patients with chronic periodontitis and analyzed gingivocrevicular fluid samples for periodontopathic bacteria by polymerase chain reaction (PCR); they also analyzed clinical parameters related to periodontitis. They found that both probing pocket depth and clinical attachment level, improved in the propolis-treated group to a statistically significant level and concluded, that propolis-based treatments, could become supportive therapeutic options for chronic periodontitis. Giammarinaro et al. (2018) evaluated in a randomized study on 40 subjects, the antioxidant capacity of a propolis and herbal formulation, as an adjunctive therapy to non-surgical periodontal treatment, without finding significant clinical differences between the two groups; however, patients in the experimental propolis group, obtained better results in terms of reduction of OS. Bretz et al. (2014) conducted a randomized clinical trial to evaluate the efficacy of a propolis rinse on induced gingivitis, in a sample of 21 pairs of twins. After 21 days without oral hygiene, they found no differences between the propolis rinse and control groups, in terms of papillary hemorrhage measurements or standard digital imaging of gum tissues. Park et al. (2021) investigated the clinical and immunological efficacy and the antioxidant and anti-inflammatory properties of propolis extracts, in a multicenter randomized controlled study, on a large sample of 104 patients with incipient gingivitis and periodontitis, and found a significant difference in the modified gingival index at 4 and 8 weeks be-tween the test and control groups; furthermore, in the test group, crevicular IL-6 was reduced and salivary metalloproteinase was increased after 8 weeks. A recent systematic review and meta-analysis of randomized clinical trials conducted by us, concluded that propolis is safe to use and can improve PD treatment outcomes, by reducing pocket probing depth (López-Valverde et al., 2021).

### 3.8 Phytohormones

#### 3.8.1 Melatonin

Melatonin is a sleep-regulating hormone produced in the pineal gland, although it can be found in plants, fungi and bacteria, such as *Tanacetumparthenium* and *Hypericumperforatum* (Arnao and Hernández-Ruiz, 2018). Murch et al. (1997) in a recent systematic review identified up to 236 plant species in which endogenous melatonin has been studied and quantified. In recent years, melatonin has been found to be a potent free radical suppressor and to possess anti-inflammatory properties (Reiter et al., 2000) and there are an abundant number of studies in the literature, *in vitro* and *in vivo*, that have investigated these properties. Gómez-FloritRamis and Monjo (2013) studied the effects of melatonin on human gingival fibroblasts for possible use in periodontal applications, which could contribute to protect and restore the integrity of gingival tissues, thus showing a potential use for the treatment of PD. Tan et al. (2021) examined *in vitro*, whether melatonin was able to induce cellular rejuvenation and found that the melatonin receptor was able to mediate the restoration of hormone-related autophagy, suggesting that melatonin could attenuate cellular senescence.

However, the results of experimental and clinical studies are contradictory. Bazyar et al. (2019), after melatonin supplementation in patients with type 2 diabetes and chronic periodontitis, found a significant reduction in IL-6 levels and significant differences in clinical attachment loss and pocket depth in the intervention and control groups. Similarly, in a randomized clinical study on a sample of seventy-four patients, the melatonin group showed a significantly greater gain in clinical attachment level and a reduction in pocket depth compared to the control group; likewise, salivary TNF-α levels were significantly lower in the melatonin group compared to the placebo group (El-Sharkawy et al., 2019). However, a combined study in subjects with periodontitis and induced periodontitis in rats by Konečná et al. (2021) found no significant effect of melatonin on alveolar bone loss, either radiographically or with micro-CT, and only gingival recession was the only macroscopic measure that improved in rats. Analysis of salivary markers of OS also revealed no beneficial effects in both rats and humans, despite clearly elevated melatonin concentrations in the melatonin-treated groups.

## 4 Discussion

**FIGURE 3:**
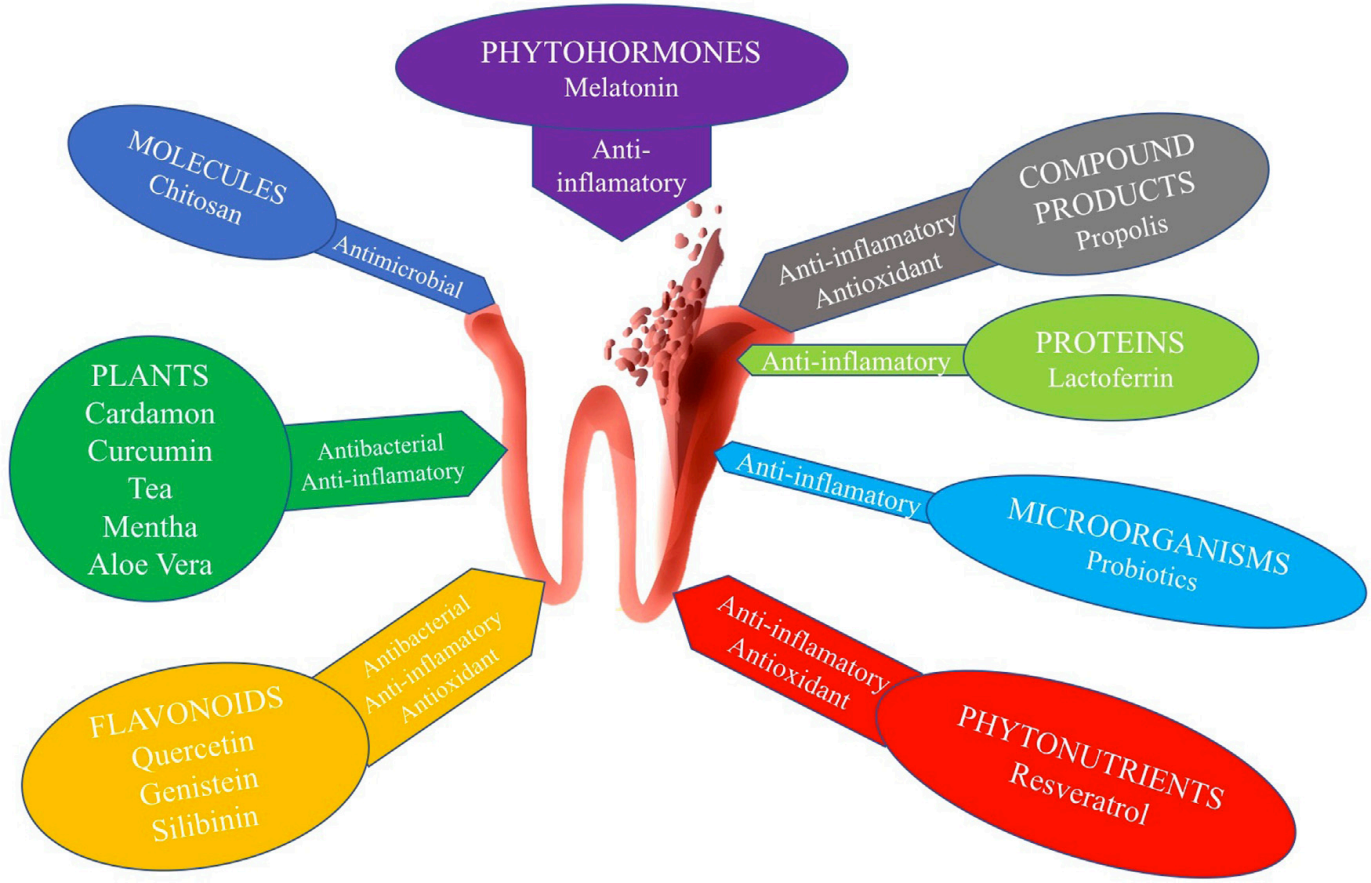
Schematic representation of the different actions of each product category.

We studied 14 natural elements as possible complementary treatments for PD, which we consider to be the most researched in the scientific literature, and classified them into eight categories, according to their specific functions (Figure 3).

Because dental plaque has a significant role in the development of PD, its treatment is critical in the therapy of these disorders. Brushing has been utilized as a means of biofilm removal since ancient times; nevertheless, it has been demonstrated that even the most modern toothbrushes are incapable of entirely eliminating bacterial plaque (Van der Weijden and Slot, 2015).

Natural products have sparked significant interest in the treatment of oral health in recent years. According to a macro-survey done in the United States, around 35% of respondents utilized natural treatments to treat oral diseases (Hoglund et al., 2020).

Within the group of molecules, chitosan has demonstrated *in vitro*, a potent effect against periodontal pathogens through a diverting effect on bacterial coaggregation, inhibiting biofilm formation, and an anti-inflammatory activity, through modulation of the fibroblastic inflammatory response (Arancibia et al., 2013; Costa et al., 2014). However, its antioxidant capacity is believed to be limited, so it is necessary to potentiate it with certain compounds (Lunkov et al., 2020; Zhao et al., 2022).

The use of phytotherapy in the treatment of PD has a wide repercussion in the scientific literature. Cardamom essential oils have been investigated as antibacterial and bacterial inhibitors, and some research has attributed to them biofilm formation inhibitory and IL-1 β, TNF- α and IL-8 reducing characteristics (Mamgain et al., 2017; Souissi et al., 2020). Turmeric has been shown to be effective as an adjuvant treatment for PD (Zhang et al., 2022a), reducing gingival indices and sulcus bleeding; however, the results of clinical studies are controversial, with findings ranging from those reporting great efficacy as an adjuvant treatment (Mohammad, 2020) to those considering it completely ineffective (Pérez-Pacheco et al., 2021). Green tea catechin has been shown *in vitro*, to be effective in the treatment of PD by suppressing specific periodontal infections (Kushiyama et al., 2009), and some clinical investigations have shown its efficiency as an adjuvant therapy for PD (Rezvani et al., 2022). There are few references to the usefulness of peppermint in the treatment of PD, and we only discovered one *in vitro* study that looked at its antibiofilm potential, in conjunction with a chitosan nanogel (Ashrafi et al., 2019). Finally, Aloe Vera has been studied as an adjuvant to non-surgical periodontal therapy in diabetic individuals with chronic periodontitis, yielding better levels of attachment (Pradeep et al., 2016).

Flavonoids have been shown *in vitro* and *in vivo*, to be effective in the prevention and treatment of periodontitis, by significantly reducing pathogenic species (Mooney et al., 2021). Quercetin has been considered an ideal therapeutic agent in the treatment of PD due to its ability to improve the OS of periodontal ligament cells as well as prevent alveolar bone resorption (Wei et al., 2021), and some studies have demonstrated *in vitro*, its ability to dampen the production of inflammatory mediators such as IL-1 β, IL-6, IL-8, and TNF- α (He et al., 2020). Similarly, for its OS attenuating impact and powerful anti-inflammatory activity, genistein and silibinin have been examined *in vivo* in the treatment of human periodontitis (Choi et al., 2016; Li et al., 2023). In fact, there is growing evidence that flavonoids have a role in type 2 diabetes, a bidirectional disease with periodontitis (Martín and Ramos, 2021).

Because of its potent anti-inflammatory action, resveratrol is thought to slow the progression of PD (Cirano et al., 2021), and some research has highlighted its important role in patients with type 2 diabetes and associated periodontitis (Zare Javid et al., 2017; Javid et al., 2019), both in the restriction of serum IL6 levels and its ability to reduce glycemia (Huang et al., 2020).

The use of probiotics in the treatment of PD would be based on the premise of oral microbiome dysbiosis and the host immunological response to it (Berlutti et al., 2011). Although there is no clear assurance, probiotics have been advocated in certain clinical trials with the goal of restoring the devastated microbiome and reducing the course of PD (Alkaya et al., 2017; Kuru et al., 2017).

Lactoferrin, for example, is thought to be bactericidal against certain periodontal pathogens and has been tested *in vitro* for its efficacy on the growth and biofilm formation of *Porphyromonas gingivalis* and *Prevotella intermedia* as adjuvant therapy for periodontal diseases (Berlutti et al., 2011), demonstrating a beneficial effect on bleeding and probing depth.

Bee-derived products, such as propolis, have piqued the interest of many researchers in recent years, owing to their exceptional antioxidant capacity, and there is a wealth of scientific evidence in the literature, reflected in numerous clinical trials and meta-analyses, demonstrating their utility in non-surgical periodontal treatment (Giammarinaro et al., 2018; Nakao et al., 2020; López-Valverde et al., 2021; Park et al., 2021). Its antihyperglycemic effectiveness in type 2 diabetes has also been demonstrated (Ochoa-Morales et al., 2022).

Finally, we considered the role of plant-derived melatonin for its anti-inflammatory properties; however, both experimental and clinical studies, yield contradictory results; while some report a significant reduction in IL-6 TNF- levels in patients with type 2 diabetes and periodontitis, other studies found no significant effects of its efficacy (Bazyar et al., 2019; Konečná et al., 2021).

## 5 Limitations, future perspectives and considerations

**FIGURE 4:**
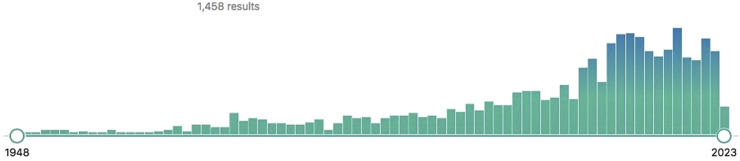
Publications in the US National Library of Medicine database through April 2023, using the MeSH terms (Periodontal Diseases/prevention & control) and (Biological Products). Source: US National Library of Medicine.

Natural product use for PD therapy has expanded significantly in recent decades, as has research in this subject. As of April 2023, a PubMed literature search utilizing the MeSH keywords [Periodontal Diseases/Prevention & Control] and [Biological Products] generated 1458 items (Figure 4).

PD is a condition with a high global occurrence that, while not deadly, impairs the quality of life of those who suffer from it, making it a burdensome illness.

Our study evaluated the existing knowledge and scientific evidence of the use of these products for the prevention and treatment of PD, assessing different routes of administration in different studies, ranging from *in vitro*, to experimental studies in different models and clinical trials. The Agency for Healthcare Research and Quality tool was used to assess the level of scientific evidence in the various studies, which ranges from the highest (meta-analysis) to the lowest (opinion of a committee of experts, clinical experience of experts or clinical experience of a respected authority) (AHRQ, 2003). We observed that the flavonoid quercetin, together with propolis, were the natural products, used in the treatment of PD, that showed the highest antioxidant, anti-inflammatory and antimicrobial activity. (Table 2).

**TABLE 2:** Scientific evidence of the antioxidant, anti-inflammatory and antimicrobial activity of the different products considered in this review.

Product	Antioxidant activity	Anti-inflammatory activity	Antimicrobial activity
Chitosan	++	++	++
Cardamom	+	+	+
Curcumin	+	++	++
Quercetin	+++	++	++
Genistein	+	+	+
Silibinin	+	+	+
Resveratrol	+	++	++
Probiotics	+	+	++
Tea	+	++	++
Lactoferrin	+	+	+++
Mentha	+	+	++
Propolis	+++	++	++
Aloe Vera	+	++	++
Melatonin	++	++	++

+Low scientific evidence; ++ Moderate scientific evidence; +++ High scientific evidence.

However, we are aware of the limitations of this review, especially the lack of homogeneity in the design of the studies, the small sample size of some of them and, above all, our grouping of the different investigations on the same product, since otherwise it would have been impossible to produce a review article and would have been the subject of a treatise in itself. It should also be noted that it is completely impossible to cover all the natural elements offered in the scientific literature for the treatment of PD, and we have limited ourselves to the most studied ones.

Taking into consideration these shortcomings, future research should consider suitable experimental models, highly predictive, since models such as the rat, are extremely resistant to periodontitis, however, the porcine and canine models are more suitable because of their similarity to the human.

On the other hand, it has been fully demonstrated that natural products have antioxidant, anti-inflammatory and antimicrobial effects and are able to help eliminate the inflammatory and oxidative response, capable of destroying the hard and soft tissues of the mouth and in the context in which the world population moves, of resistance to certain antimicrobials and the increase of adverse reactions, the development of new therapeutic systems for the treatment of chronic diseases such as PD should be taken into account by researchers. In fact, a meta-analysis of clinical studies, recently carried out by us, on the use of metronidazole as an adjuvant treatment for peri-implantitis (a pathology with certain similarities to periodontitis), found no definitive conclusions on its effect. (López-Valverde et al., 2023).

Therefore, we believe that further studies are needed to define the therapeutic effect of these products and to determine the appropriate amounts of natural extracts, for the correct design of clinical trials.
